# Effect of posterior pericardiotomy in cardiac surgery: A systematic review and meta-analysis of randomized controlled trials

**DOI:** 10.3389/fcvm.2022.1090102

**Published:** 2022-12-23

**Authors:** Giovanni Jr Soletti, Roberto Perezgrovas-Olaria, Lamia Harik, Mohamed Rahouma, Arnaldo Dimagli, Talal Alzghari, Michelle Demetres, Brenden A. Bratton, Mohammad Yaghmour, Divyaam Satija, Christopher Lau, Leonard N. Girardi, Tomas A. Salemo, Mario Gaudino

**Affiliations:** ^1^Department of Cardiothoracic Surgery, Weill Cornell Medicine, New York, NY, United States; ^2^Samuel J. Wood Library and C.V. Starr Biomedical Information Center, Weill Cornell Medicine, New York, NY, United States; ^3^Division of Cardiothoracic Surgery, University of Miami Miller School of Medicine and Jackson Memorial Hospital, Miami, FL, United States

**Keywords:** cardiac surgery, posterior pericardiotomy, postoperative atrial fibrillation, pericardial effusion, meta-analysis

## Abstract

**Background:**

Posterior pericardiotomy (PP) has been shown to reduce the incidence of pericardial effusion and postoperative atrial fibrillation (POAF) after cardiac surgery. However, the procedure and the totality of its effects are poorly known in the cardiac surgery community. We performed a study-level meta-analysis of randomized controlled trials (RCTs) to evaluate the impact of PP in cardiac surgery patients.

**Methods:**

A systematic literature search was conducted on three medical databases (Ovid MEDLINE, Ovid Embase, Cochrane Library) to identify RCTs reporting outcomes of patients that received a PP or no intervention after cardiac surgery. The primary outcome was the incidence of POAF. Key secondary outcomes were operative mortality, incidence of pericardial and pleural effusion, cardiac tamponade, length of stay (LOS), pulmonary complications, amount of chest drainage, need for intra-aortic balloon pump, and re-exploration for bleeding.

**Results:**

Eighteen RCTs totaling 3,531 patients were included. PP was associated with a significantly lower incidence of POAF (odds ratio [OR] 0.45, 95% confidence interval [CI] 0.32–0.64, *P* < 0.0001), early (OR 0.18, 95% CI 0.10–0.34, *P* < 0.0001) and late pericardial effusion (incidence rate ratio 0.13, 95% CI 0.06–0.29, *P* < 0.0001), and cardiac tamponade (risk difference −0.02, 95% CI −0.04 to −0.01, *P* = 0.001). PP was associated with a higher incidence of pleural effusion (OR 1.42, 95% CI 1.06–1.90, *P* = 0.02), but not pulmonary complications (OR 0.82, 95% CI 0.56–1.19; *P* = 0.38). No differences in other outcomes, including operative mortality, were found.

**Conclusions:**

PP is a safe and effective intervention that significantly decreases the incidence of POAF and pericardial effusion following cardiac surgery.

**Systematic review registration:**

https://www.crd.york.ac.uk/prospero/display_record.php?RecordID=261485, identifier: CRD42021261485.

## 1. Introduction

Despite advances in postsurgical management, postoperative atrial fibrillation (POAF) still represents the most frequent complication following cardiac surgery, resulting in a substantial clinical and economic burden (1–3). An important trigger of POAF, among others, seems to be the accumulation of fluid in the posterior pericardium (4, 5). Since its introduction in 1995, posterior pericardiotomy (PP) has been hypothesized to reduce the incidence of POAF and pericardial effusion by means of an incision in the posterior pericardium, allowing pericardial fluid to drain into the left pleural space (6).

Over the past two decades, multiple randomized controlled trials (RCTs) have tested this intervention, providing data on its high efficacy in reducing POAF (7–24). However, the procedure and the totality of its benefits and safety profile are poorly known in the cardiac surgery community.

We conducted a systematic review and study-level meta-analysis to evaluate the outcomes of patients that received a PP in addition to cardiac surgery compared to patients that received no additional intervention.

## 2. Methods

This review was registered with the National Institute for Health Research International Registry of Systematic Reviews (PROSPERO; CRD42021261485). The manuscript follows the Preferred Reporting Items for Systematic Reviews and Meta-Analyses (PRISMA) guideline (25).

### 2.1. Search strategy

A qualified librarian (MD) performed a systematic literature search to identify potential studies comparing the outcomes of patients that received cardiac surgical procedures and PP to patients that received a cardiac surgical procedure and no PP. Searched were originally run on July 2021 and updated on December 28, 2021 using the following databases (Ovid MEDLINE, Ovid EMBASE, and The Cochrane Library) from inception to present. The search strategy for Ovid MEDLINE is available in Supplementary Table 1.

### 2.2. Study selection and data extraction

After deduplication, title and abstracts of the remaining articles were screened against predefined inclusion and exclusion criteria by two authors (GS and RP-O) independently. Any discrepancies were adjudicated by the senior author (MG). All relevant English-written RCTs reporting outcomes of adult patients (≥18 years old) undergoing open heart surgery with and without a concomitant PP procedure were considered for inclusion. All the studies that were not RCTs were excluded. The full text of the selected manuscripts was retrieved for a second round of screening. The references were also reviewed for pertinent studies not identified through the initial search. The quality assessment of the included RCTs was performed using The Cochrane Collaboration's Risk of Bias 2 (RoB 2) tool for randomized trials (26).

The PP procedure was defined as any incision in the posterior pericardium allowing drainage of the pericardial cavity into the left pleural space, with or without the insertion of a chest tube in the posterior pericardial space. A detailed description of the steps to perform a PP has been previously published (27).

Two authors (GS and RP-O) separately performed data extraction and the accuracy was verified by the senior investigatto (MG). The following variables were extracted from each RCT: study characteristics (first author, year of publication, publishing journal, country, type of cardiac surgery, and sample size), patient demographics (age, sex, smoking status, hypertension, diabetes, and dyslipidemia), and key outcomes.

### 2.3. Outcomes

The primary outcome was the incidence of POAF. The secondary outcomes were operative mortality, early and late pericardial effusion, cardiac tamponade, pleural effusion, amount of total chest drainage (mediastinal plus pleural drainage), duration of intensive care unit (ICU) and hospital length of stay (LOS), pulmonary complications, need for intra-aortic balloon pump (IABP), and re-exploration for bleeding. For the secondary outcomes, individual study definitions were used.

### 2.4. Statistical analysis

The number of events of short-term outcomes was extracted for each group and pooled with an inverse variance method and described as odds ratio (OR) with 95% confidence interval (CI). When both groups reported zero events, risk difference (RD) was used as pooled estimate.

For the only follow-up outcome (late pericardial effusion), we took into account the variability in the lengths of follow-up in each study and therefore incidence rate ratio (IRR) were pooled for this outcome. IRRis the ratio of the number of events and the number of patient-years. Inverse variance method with both fixed- and random-effect models was used to pool this estimate.

The standardized mean difference (SMD) with 95% CI was used to compare chest drainage, as well as ICU and hospital LOS between patients with and without PP.

The I^2^ was used to evaluate statistical heterogeneity that is the proportion of the variability in the estimates due to heterogeneity rather than by chance. A value of 25, 50, and 75% identified low, moderate and high heterogeneity, respectively. Egger's test and inspection of funnel plot was used to assess the presence of small-study effect.

A leave-one-out approach was used as sensitivity analysis for the primary outcome: the meta-analytic estimates were recalculated by excluding one study per time. Also, meta-regression was performed by regressing the estimates against the preoperative characteristics (age, female sex, hypertension, dyslipidemia, smoking, and diabetes), and the type of surgery (coronary artery bypass grafting [CABG], aortic valve replacement [AVR], or other valve surgery).

In all analyses, the control group was the reference group. Statistical analyses were performed in R (version 4.2.0; R Project; R Foundation for Statistical Computing, Vienna, Austria) using the packages: meta, dmetar. A *P*-value < 0.05 was used as threshold for statistical significance.

## 3. Results

### 3.1. Study characteristics

Of the 4,017 screened articles, 18 articles published between 1997 and 2021 met our inclusion criteria and were included in the present analysis (7–24). The PRISMA flow diagram outlining the study selection process is provided in Supplementary Figure 1.

Assessment of study quality using the RoB 2 tool showed that all but four (15, 17, 18, 21) RCTs had an unclear risk of bias regarding allocation concealment and blinding of researchers and participants. Details of the quality assessment are provided in Supplementary Table 2.

Ten (55.5%) of the included RCTs were conducted in Turkey, three (16.6%) in Iran, while China, Egypt, England, Thailand, and the United States contributed with one RCT (5.6%) each. Thirteen (72.2%) RCTs included patients undergoing isolated CABG, three (16.7%) enrolled patients undergoing either isolated CABG or CABG with valve surgery, one (5.6%) RCT enrolled patients undergoing valve and/or aortic surgery, and one (5.6%) included CABG, AVR, and aortic surgery patients. Characteristics of the included RCTs are provided in Table 1.

**Table 1:** Summary of the included RCTs.

**References**	**Journal (2020 IF)**	**Country**	**Type of procedure**	**Sample size** **(men, %)**	**No of patients** **per arm**	**Outcomes and results**
Arbatli et al. (7)	*The Journal of Cardiovascular Surgery (1.888)*	Turkey	CABG	113 (89, 79%)	PP: 54 Control: 59	**Main outcomes assessed** POAF, pericardial effusion, pleural effusion **Assessment modalities** Continuous telemetry + EKG, echocardiography, chest X-rays **Main findings** No difference in POAF between PP and control groups (*P =* 0.32). The incidence of POAF was higher in patients with mild to moderate compared to those with no or minimal pericardial effusion (*P =* 0.017). Pericardial effusion was lower in the PP group (*P =* 0.02). No significant difference in pleural effusion between groups.
Asimakopoulos et al. (8)	*The Journal of Thoracic and Cardiovascular Surgery (5.209)*	UK	CABG	100 (NR)	PP: 50 Control: 50	**Main outcomes assessed** POAF **Assessment modalities** Continuous telemetry + EKG **Main findings** No significant difference in the incidence of AF between groups.
Bakhshandeh et al. (9)	*Asian Cardiovascular and Thoracic Annals (0.49)*	Iran	CABG/ Valve surgery	410 (164, 40%)	PP: 205 Control: 205	**Main outcomes assessed** POAF, pericardial effusion **Assessment modalities** Not stated for POAF, echocardiography **Main findings** No significant difference in POAF between groups. At all time points, the majority of patients who underwent PP were free of effusion, but none of those in the control group were free of effusion (*P < * 0.05).
Cakalagaoglu et al. (10)	*The Heart Surgery Forum (0.676)*	Turkey	CABG/ Valve surgery	100 (83, 83%)	PP: 50 Control: 50	**Main outcomes assessed** POAF, pericardial effusion **Assessment modalities** Continuous telemetry + EKG, echocardiography, chest X-ray **Main findings** No significant difference in POAF. Before discharge, the control group had a significantly higher number of patients with moderate, large, and very large pericardial effusions compared with the PP group.
Ekim et al. (11)	*Medical Science Monitor (2.649)*	Turkey	CABG	100 (65, 65%)	PP: 50 Control: 50	**Main outcomes assessed** POAF, pericardial effusion, pleural effusion **Assessment modalities** Continuous telemetry + EKG, echocardiography. Not stated for pleural effusion. **Main findings** Early pericardial effusion was significantly lower in the PP group (*P =* 0.0001). The number of patients who developed POAF was significantly lower in the PP group compared with the control group (10 vs. 30%, *P < * 0.01). No difference in the incidence of pleural effusion was found.
Erdil et al. (12)	*Journal of Cardiac Surgery (1.62)*	Turkey	Valve surgery/ Aortic	100 (39, 39%)	PP: 50 Control: 50	**Main outcomes assessed** Pericardial effusion, pleural effusion **Assessment modalities** Echocardiography. Not stated for pleural effusion. **Main findings** Early pericardial effusion developed in 4/50 (8%) patients of the PP group and in 19/50 (38%) of the control group (*P < * 0.001). No late pericardial effusion in the PP group, 9/50 (18%) in control group (*P < * 0.003). No significant difference in the incidence of pleural effusion between groups.
Farsak et al. (13)	*European Journal of Cardio-Thoracic Surgery (4.191)*	Turkey	CABG	150 (51, 34%)	PP: 75 Control: 75	**Main outcomes assessed** POAF, pericardial effusion, pleural effusion **Assessment modalities** Continuous telemetry + EKG, echocardiography. Not stated for pleural effusion. **Main findings** POAF developed in 7 patients (9.3%) in the PP group and 24 patients (32%) in the control group (*P < * 0.001). Early pericardial effusion developed in 42.6% (32/75) of the control group and in 10.6% (8/75) of the PP group (*P < * 0.0001). No late pericardial effusion developed in the PP group, while 7 (9.3%) developed in the control group (*P < * 0.013). No significant difference in pleural effusion.
Fawzy et al. (14)	*Interactive CardioVascular and Thoracic Surgery (1.905)*	Egypt	CABG	200 (132, 66%)	PP: 100 Control: 100	**Main outcomes assessed** POAF, pericardial effusion **Assessment modalities** Continuous telemetry + ECG, echocardiography. **Main findings** The incidence of POAF was significantly lower in the PP group than in the control group (13 vs. 30%, *P =* 0.01). Postoperative pericardial effusion was significantly lower in the PP group (15 vs. 50 patients, *P =* 0.04).
Gaudino et al. (15)	*Lancet (79.321)*	USA	CABG/ AVR/ Aortic surgery	420 (318, 76%)	PP: 212 Control: 208	**Main outcomes assessed** POAF, pericardial effusion, pleural effusion **Assessment modalities** Continuous telemetry and daily EKG, echocardiography, chest X-rays. CT scan in case of moderate-large pericardial effusion. **Main findings** POAF in PP group 37/212 (18%) compared to 66/208 (32%) in the no intervention group (aOR 0.44, 95% CI: 0.27–0.70; *P < * 0.0005). Pericardial effusion in PP 27/212 (12%) vs. 44/208 (21%) in the control group (RR 0.58, 95% CI: 0.37–0.91). No significant difference regarding pleural effusion.
Haddadzadeh et al. (16)	*Acta Medica Iranica (0.26)*	Iran	OPCABG	207 (142, 69%)	PP: 105 Control: 102	**Main outcomes assessed** POAF, pericardial effusion **Assessment modalities** Continuous telemetry + EKG, echocardiography **Main findings** No significant difference between the two groups regarding POAF or pericardial effusion.
Kaya et al. (17)	*Kardiochirurgia i Torakochirurgia Polska (0.23)*	Turkey	CABG	96 (77, 80%)	PP: 30 Control: 66	**Main outcomes assessed** POAF, pericardial effusion **Assessment modalities** Continuous telemetry + EKG, echocardiography **Main findings** No significant differences were found between the groups regarding POAF (*P =* 0.392). The incidence of moderate to severe pericardial effusion in PP group was significantly lower than in the other groups on the 30th post-operative day (*P =* 0.028).
Kaya et al. (18)	*Interactive CardioVascular and Thoracic Surgery (1.905)*	Turkey	CABG	142 (118, 83%)	PP: 70 Control: 72	**Main outcomes assessed** POAF, pericardial effusion, pleural effusion **Assessment modalities** Portable EKG telemetry, echocardiography. Not stated for pleural effusion **Main findings** POAF occurred in 27.78% of the cases in the open group and 8.57% of the patients in the closure group (*P =* 0.003). Difference in pericardial effusion favored the closure group (*P =* 0.039). No significant difference in pleural effusion between groups.
Kaya et al. (19)	*Thoracic and Cardiovascular Surgeon (1.827)*	Turkey	CABG	210 (164, 78%)	PP: 103 Control: 107	**Main outcomes assessed** POAF, pericardial effusion, pleural effusion **Assessment modalities** Portable EKG telemetry, echocardiography. Not stated for pleural effusion **Main findings** Statistically significant results were obtained in the amount of PE (*P =* 0.034 on POD 2; *P =* 0.019 on POD 5) and POAF (*P =* 0.019) in favor of the study group. No significant difference regarding pleural effusion.
Kaygin et al. (20)	*The Tohoku Journal of Experimental Medicine (1.848)*	Turkey	CABG	415 (212, 51%)	PP: 213 Control: 212	**Main outcomes assessed** POAF, pericardial effusion, pleural effusion **Assessment modalities** Not stated for POAF and pleural effusion. Echocardiography. **Main findings** POAF (*P < * 0.0001), early (*P < * 0.001) and late pericardial effusion (*P < * 0.0001) occurred more frequently in the control group compared with the PP group. PP was associated with an increase in pleural effusion requiring intervention (*P =* 0.002).
Kongmalai et al. (21)	*Journal of the Medical Association of Thailand (0.09)*	Thailand	CABG	20 (10, 50%)	PP: 10 Control: 10	**Main outcomes assessed** POAF, pericardial effusion, pleural effusion **Assessment modalities** Continuous telemetry + EKG, echocardiography, chest X-rays. **Main findings** No significant differences in POAF (*P =* 1) and early pericardial effusion (*P =* 1). The incidence of pleural effusion was higher in the PP group (*P =* 0.028).
Kuralay et al. (22)	*The Journal of Thoracic and Cardiovascular Surgery (5.209)*	Turkey	CABG	200 (150, 75%)	PP: 100 Control: 100	**Main outcomes assessed** POAF, pericardial effusion, pleural effusion **Assessment modalities** Continuous telemetry + EKG, echocardiography, Not stated for pleural effusion. **Main findings** POAF developed in 6 patients (6%) in PP group and in 34 patients (34%) in the control group (*P =* 0.0000007). The incidence of early and late pericardial effusion was significantly more frequent in the control group (*P < * 0.001 for both). No statistically significant difference was found regarding pleural effusion.
Sadeghpour et al. (23)	*Multidisciplinary Cardiovascular Annals (NA)*	Iran	CABG	80 (63, 79%)	PP: 40 Control: 40	**Main outcomes assessed** Pericardial effusion **Assessment modalities** Echocardiography **Main findings** Early pericardial effusion was more frequent in the control group (45 vs. 15%; *P =* 0.01). Late pericardial effusion was also more frequent in the control group (57 vs. 15%; *P =* 0.01).
Zhao et al. (24)	*Journal of International Medical Research (1.671)*	China	CABG/ Valve surgery	458 (263, 57%)	PP: 228 Control: 230	**Main outcomes assessed** POAF, pericardial effusion, pleural effusion **Assessment modalities** Not stated for POAF. Echocardiography **Main findings** The incidence of POAF in the PP group was significantly lower compared with the control group (*P =* 0.044). The incidence of small (*P =* 0.004) and moderate-to-large (*P =* 0.02) pericardial effusion in the PP group was significantly lower than in the control group. The incidence of moderate-to-large pleural effusion in the PP group was significantly higher than in the control group (*P =* 0.015).

aOR, adjusted odds ratio; AVR, aortic valve replacement; CABG, coronary artery bypass grafting; CT, computerized tomography; EKG, electrocardiography; IF, impact factor; NA, not available; NR, not reported; OPCABG, off-pump coronary artery bypass grafting; PO, postoperative day; POAF, postoperative atrial fibrillation; PP, posterior pericardiotomy; RCT, randomized controlled trial; SD, standard deviation.

### 3.2. Patient characteristics

A total of 3,531 patients were pooled in the analysis. The number of patients in the included RCTs ranged from 20 to 458, with a median sample size of 146 (interquartile range: 100–209). Overall, 1,745 (49.4%) patients received a PP and 1,786 (50.6%) underwent cardiac surgery without PP.

Men represented 62.2% of the studied population (62.8 and 61.7% of the PP and control groups, respectively). The mean age range was 40.9 to 67.3 years in the PP group and 43.2 to 68.2 years in the control group. The prevalence of diabetes ranged from 17.3 to 65% in the PP group and from 10 to 56.9% in the control group. The prevalence of dyslipidemia ranged from 36 to 75% in the PP group and from 35.3 to 71.2% in the control group. The prevalence of smoking ranged from 0 to 76.1% in the PP group and from 20 to 74% in the control group. The prevalence of hypertension ranged from 20 to 80% in the PP group and from 36 to 90% in the control group.

### 3.3. Meta-analysis

**Figure 1:**
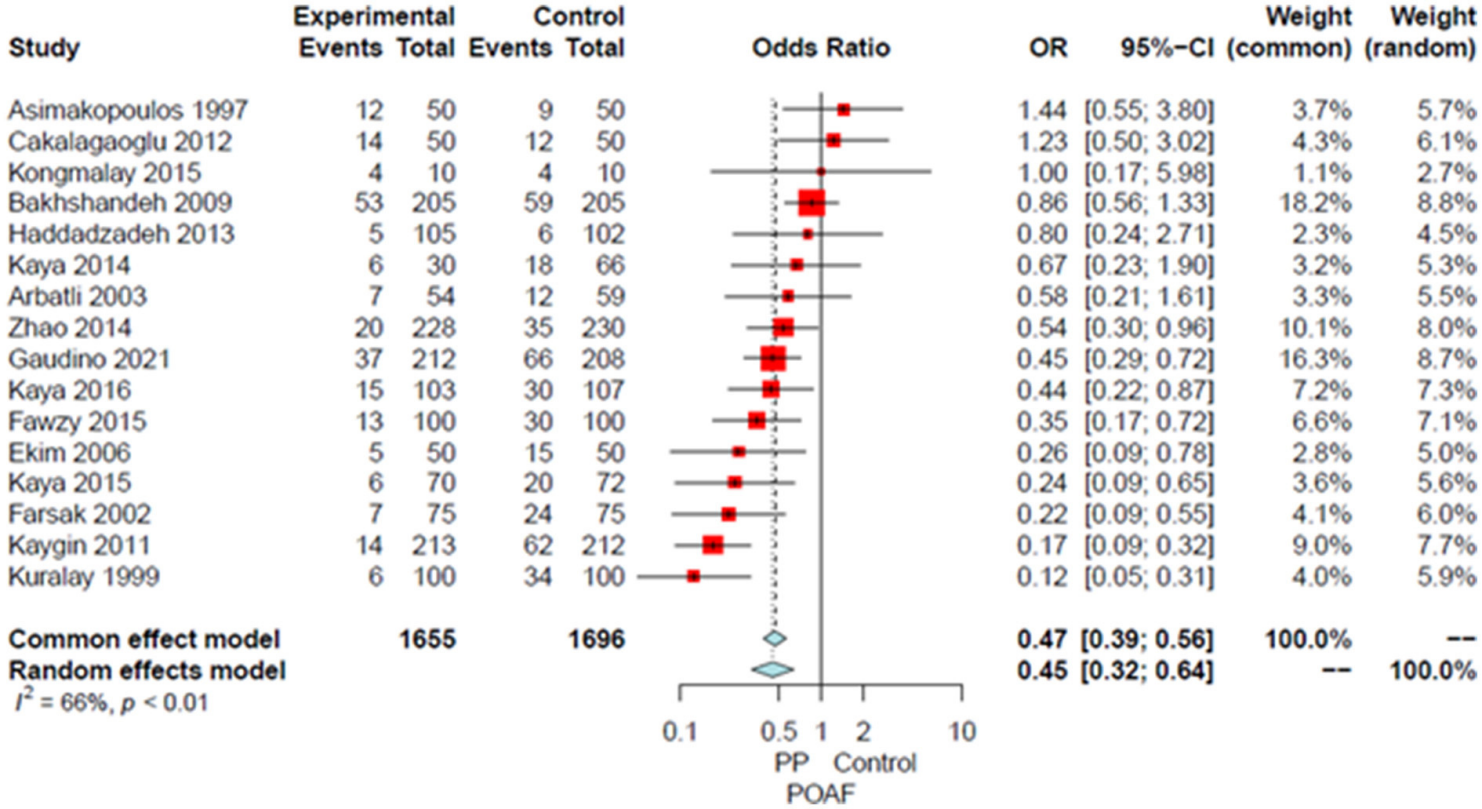
Forest plot for postoperative atrial fibrillation. CI, confidence interval; OR, odds ratio; POAF, postoperative atrial fibrillation; PP, posterior pericardiotomy.

**Figure 2:**
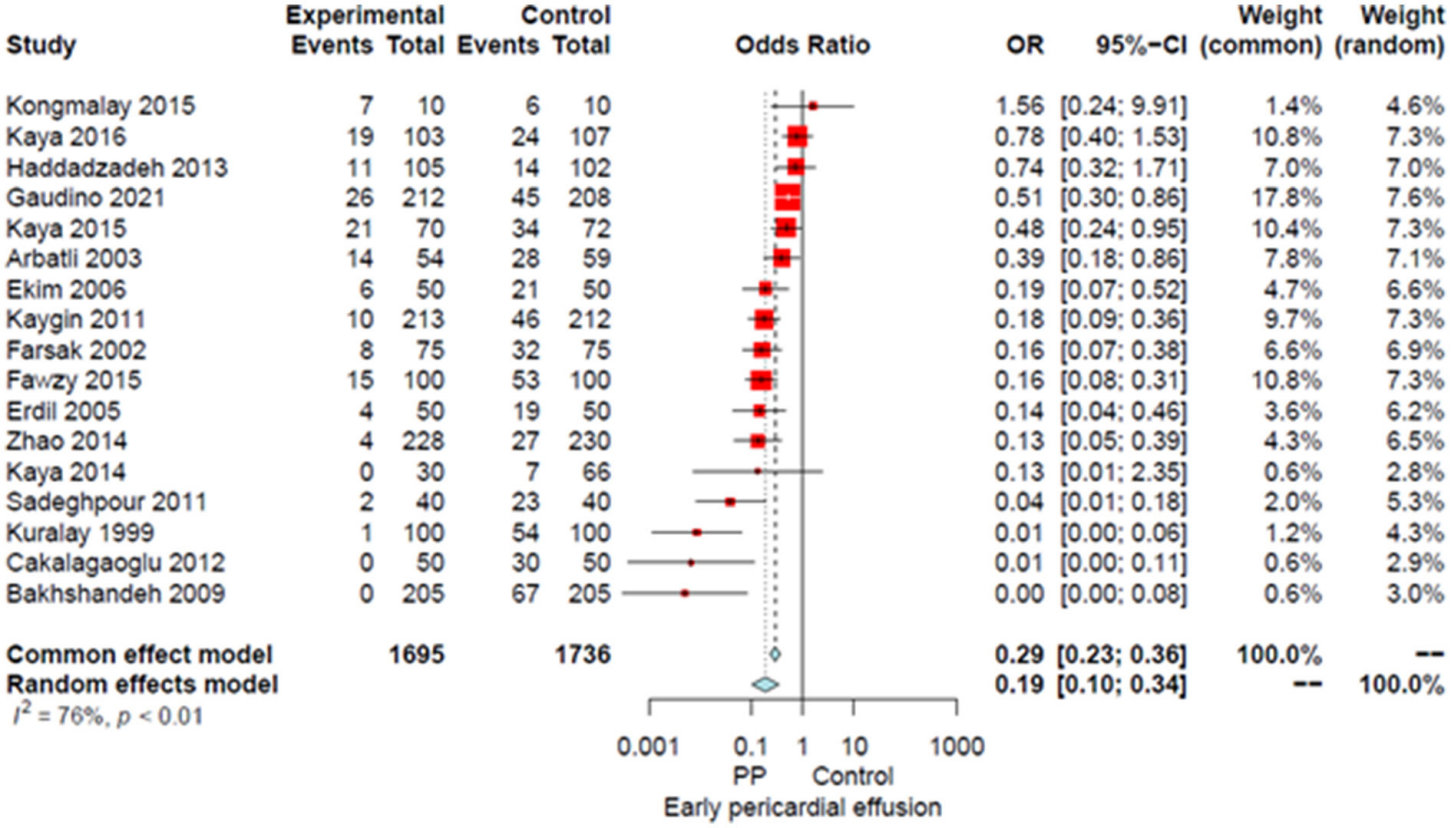
Forest plot for early pericardial effusion. CI, confidence interval; OR, odds ratio; PP, posterior pericardiotomy.

**Figure 3:**
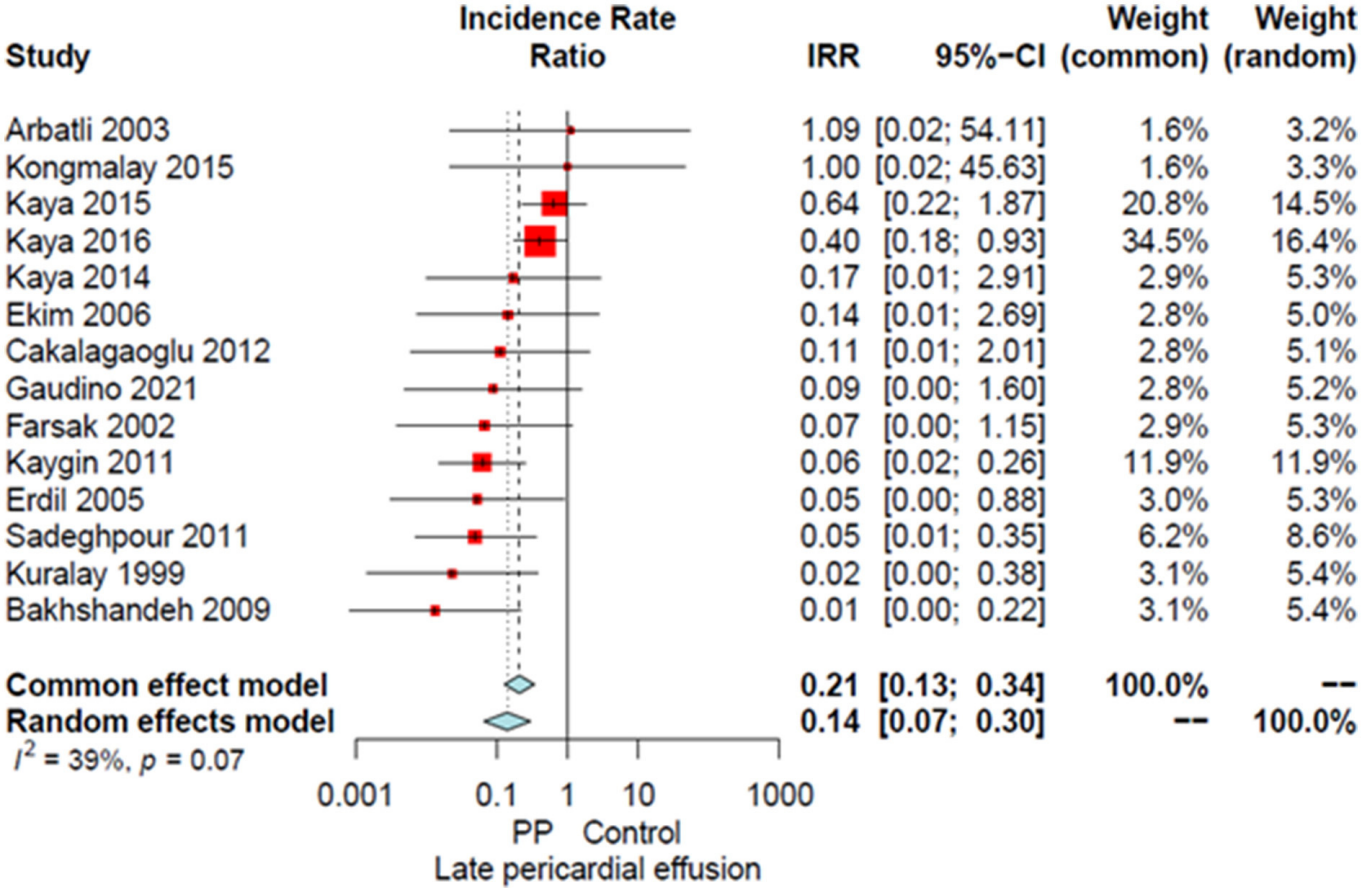
Forest plot for late pericardial effusion. CI, confidence interval; IRR, incidence rate ratio; PP, posterior pericardiotomy.

Compared to the no intervention group, patients with a PP had a significantly lower risk of POAF (OR 0.45, 95% CI 0.32–0.64, *P* < 0.0001; Figure 1), early pericardial effusion (OR 0.19, 95% CI 0.10–0.34, *P* < 0.0001; Figure 2), late pericardial effusion (IRR 0.14, 95% CI 0.07–0.30, *P* < 0.0001; Figure 3), and cardiac tamponade (RD −0.02, 95% CI −0.04 to −0.01, *P* = 0.001; Supplementary Figure 2). Patients with a PP had a higher risk of pleural effusion (265/1,165, 22.7%) compared to the no intervention group (203/1,173, 17.3%) (OR 1.42, 95% CI 1.06–1.90, *P* = 0.02; Supplementary Figure 3).

The leave-one-out analysis confirmed the solidity of the main analysis (Supplementary Figure 4).

No difference in operative mortality, pulmonary complications (84/1,168 [7.2%] in the PP group vs. 107/1,205 [8.9%] in the control group), need for IABP, re-exploration for bleeding, ICU LOS, hospital LOS, or chest drainage (range/mean volume in PP group: 450–1,421 ml/746 ml; range/mean volume in control group: 266–1,153 ml/696 ml) was found between groups (Supplementary Figures 5–11). A summary of all the outcomes and their reporting in each of the included RCTs are provided in Table 2 and Supplementary Table 3, respectively.

**Table 2:** Summary of the primary and key secondary outcomes.

**Outcome**	**No. of studies**	**Events**	**Patients**	**Effect estimate (95% CI), *P*-value**	**Heterogeneity (I^2^, *P*-value)**
POAF	16	660	3,351	OR = 0.45 (0.32–0.64), *P < * 0.0001	65.8%, *P < * 0.001
Operative mortality	11	33	2,123	RD = −0.002 (−0.01 to 0.01), *P =* 0.66	0.0%, *P =* 0.99
Early pericardial effusion	17	678	3,431	OR = 0.19 (0.10–0.34), *P < * 0.0001	76.3%, *P < * 0.001
Late pericardial effusion	14	-	2,566	IRR = 0.14 (0.07–0.30), *P < * 0.0001	38.5%, *P =* 0.07
Chest drainage	14	-	2,019	SMD = 0.10 (−0.13 to 0.34), *P =* 0.4	86.3%, *P < * 0.001
Cardiac tamponade	15	62	3,144	RD = −0.02 (−0.04 to −0.01), *P =* 0.001	55.5%, *P =* 0.01
Pleural effusion	11	468	2,338	OR = 1.42 (1.06–1.90), *P =* 0.02	38.4%, *P =* 0.09
Hospital LOS^*^	10	-	1,641	SMD = −0.11 (−0.29 to 0.06), *P =* 0.21	60.7%, *P =* 0.01
ICU LOS^*^	6	-	1,243	SMD = 0.06 (−0.15 to 0.27), *P =* 0.57	62.9%, *P =* 0.02
Pulmonary complications	12	191	2,373	OR = 0.82 (0.56–1.19), *P =* 0.30	7.1%, *P =* 0.38
Need for IABP	9	105	2,096	RD = 0.003 (−0.01 to 0.02), *P =* 0.62	0.0%, *P =* 0.97
Re-exploration for bleeding	14	100	2,944	OR = 0.78 (0.52 to 1.19), *P =* 0.25	0.0%, *P =* 0.93

^*^Measured in days. CI, Confidence interval; IABP, intra-aortic balloon pump; ICU, intensive care unit; IRR, incidence rate ratio; LOS, length of stay; OR, odds ratio; POAF, postoperative atrial fibrillation; RD, risk difference; SMD, standardized mean difference.

No evidence of publication bias was observed based on the Egger's intercept test (*P* = 0.75) (Supplementary Figure 12).

### 3.4. Meta-regression

Meta-regression failed to identify any significant association between the tested variables and the OR for the POAF (Table 3).

**Table 3:** Results of meta-regression for the primary outcome.

**Variables**	**Beta ±SE, *P*-value**
Age	0.0675 ± 0.0486, *P =* 0.17
Female sex	−0.0039 ± 0.0116, *P =* 0.74
Diabetes	−0.0105 ± 0.0120, *P =* 0.39
Dyslipidemia	0.0146 ± 0.0474, *P =* 0.76
Smoking	−0.0178 ± 0.0157, *P =* 0.26
Hypertension	0.0141 ± 0.0133, *P =* 0.29
CABG	−0.0099 ± 0.0102, *P =* 0.33
Valve surgery	0.0125 ± 0.0084, *P =* 0.14
Aortic valve replacement	0.0001 ± 0.0153, *P =* 0.99

CABG, coronary artery bypass grafting; SE, standard error.

## 4. Discussion

This meta-analysis of 18 studies found that patients with PP had a significantly lower incidence of POAF, early and late pericardial effusion, and cardiac tamponade; there was a significantly higher incidence of pleural effusion, but not an increased risk of pulmonary complications. No other differences in outcomes were found.

POAF is the most frequent complication following cardiac surgery, occurring in approximately one third of the patients (1, 28). POAF has been associated with extended postoperative LOS and increased hospital costs (28), as well as with major adverse postoperative outcomes including renal and heart failure, stroke, and mortality (4, 28, 29). Despite many attempts with medical therapy to prevent POAF, its incidence remains high (30). PP provides a safe and virtually zero-cost surgical alternative for the prevention of POAF. Notably, there has been only one report of complications related to PP (graft herniation) (31), and no there are no reports on damage to the phrenic nerve or the esophagus during PP. None of the studies included in this meta-analysis reported phrenic nerve or esophageal injuries.

Since the procedure was first described by Mulay et al. (6), several RCTs have tried to shed light on the relationship between PP and POAF (7–11, 13–22, 24, 32). However, most of these studies were limited in methodological quality and inadequately powered to yield statistically significant results. This prompted our group to perform the first high-quality, adequately powered RCT on the effect of posterior pericardiotomy on POAF, the Posterior Left Pericardiotomy for the prevention of AtriaL Fibrillation after Cardiac Surgery (PALACS) trial (15), which included 420 cardiac surgery patients undergoing CABG, AVR, and/or aortic surgery, notably excluding mitral and tricuspid surgeries.

In the PALACS trial, we found a significantly lower incidence of POAF among patients randomized to PP (17 vs. 32%, *P* = 0.0007), and a lower incidence of postoperative pericardial effusion in the PP group (12 vs. 21%, relative risk 0.58, 95% CI 0.37–0.91), but no difference in the incidence of cardiac tamponade or pleural effusion was found. In this meta-analysis, both outcomes reached statistical significance, with the incidence of cardiac tamponade being lower in the PP group and the incidence of pleural effusion being higher in PP patients. An important finding of the present analysis is that despite the higher incidence of pleural effusion, patients with PP did not have an increased risk of pulmonary complications.

This study has the following limitations. Although our systematic review identified the best available evidence evaluating the impact of PP on postoperative outcomes, the present study cannot control for individual biases of the included studies. Additionally, there was variability in POAF detection methods, perioperative management, PP technique, and in the definition and reporting of outcomes of interest. More importantly, clinical outcomes of relevance to POAF like stroke and transient ischemic attack (TIA) were not reported in most studies (88.9%) and could not be pooled for analysis.

To conclude, our meta-analysis of 18 studies found that PP is associated with a lower incidence of POAF, pericardial effusion, and cardiac tamponade, but increased incidence of pleural effusion.

## Data availability statement

The original contributions presented in the study are included in the article/Supplementary material, further inquiries can be directed to the corresponding authors.

## Author contributions

GS, MG, and TS : concept and design. GS and MD: systematic search. GS and RP-O: drafting the article. MR, AD, and MG: statistics. GS, RP-O, BB, MY, and DS: data collection. All authors analyzed the data interpretation, critical revision of the article, and approval of the article.

## References

[1] LaPar DJ Speir AM Crosby IK Fonner E Brown M Rich JB . Postoperative atrial fibrillation significantly increases mortality, hospital readmission, and hospital costs. Ann Thorac Surg. (2014) 98:527–33. 10.1016/j.athoracsur.2014.03.039 25087786

[2] Greenberg JW Lancaster TS Schuessler RB Melby SJ. Postoperative atrial fibrillation following cardiac surgery: A persistent complication. Eur J Cardiothorac Surg. (2017) 52:665–72. 10.1093/ejcts/ezx039 28369234

[3] Taha A Nielsen SJ Bergfeldt L Ahlsson A Friberg L Björck S . New-onset atrial fibrillation after coronary artery bypass grafting and long-term outcome: A population-based nationwide study from the SWEDEHEART registry. J Am Heart Assoc. (2021) 10:e017966. 10.1161/JAHA.120.017966 33251914 PMC7955471

[4] St-Onge S Perrault LP Demers P Boyle EM Gillinov AM Cox J . Pericardial blood as a trigger for postoperative atrial fibrillation after cardiac surgery. Ann Thorac Surg. (2018) 105:321–8. 10.1016/j.athoracsur.2017.07.045 29174782

[5] Gaudino M Di Franco A Rong LQ Cao D Pivato CA Soletti GJ . Pericardial effusion provoking atrial fibrillation after cardiac surgery: JACC review topic of the week. J Am Coll Cardiol. (2022) 79:2529–39. 10.1016/j.jacc.2022.04.029 35738715

[6] Mulay A Kirk AJ Angelini GD Wisheart JD Hutter JA. Posterior pericardiotomy reduces the incidence of supra-ventricular arrhythmias following coronary artery bypass surgery. Eur J Cardio-Thorac Surg. (1995) 9:150–2. 10.1016/S1010-7940(05)80063-6 7786532

[7] Arbatli H Demirsoy E Aytekin S Rizaoglu E Unal M Yagan N . The role of posterior pericardiotomy on the incidence of atrial fibrillation after coronary revascularization. J Cardiovasc Surg (Torino). (2003) 44:713–7. 14735032

[8] Asimakopoulos G Della Santa R Taggart DP. Effects of posterior pericardiotomy on the incidence of atrial fibrillation and chest drainage after coronary revascularization: A prospective randomized trial. J Thorac Cardiovasc Surg. (1997) 113:797–9. 10.1016/S0022-5223(97)70242-3 9104993

[9] Bakhshandeh AR Salehi M Radmehr H Sattarzadeh R Nasr AR Sadeghpour AH. Postoperative pericardial effusion and posterior pericardiotomy: Related? Asian Cardiovasc Thorac Ann. (2009) 17:477–9. 10.1177/0218492309341787 19917788

[10] Cakalagaoglu C Koksal C Baysal A Alici G Ozkan B Boyacioglu K . The use of posterior pericardiotomy technique to prevent postoperative pericardial effusion in cardiac surgery. Heart Surg Forum. (2012) 15:E84–89. 10.1532/HSF98.20111128 22543342

[11] Ekim H Kutay V Hazar A Akbayrak H Başel H Tuncer M. Effects of posterior pericardiotomy on the incidence of pericardial effusion and atrial fibrillation after coronary revascularization. Med Sci Monit Int Med J Exp Clin Res. (2006) 12:CR431–434. 17006403

[12] Erdil N Nisanoglu V Kosar F Erdil FA Cihan HB Battaloglu B. Effect of posterior pericardiotomy on early and late pericardial effusion after valve replacement. J Card Surg. (2005) 20:257–60. 10.1111/j.1540-8191.2005.200375.x 15854088

[13] Farsak B Günaydin S Tokmakoglu H Kandemir O Yorgancioglu C Zorlutuna Y. Posterior pericardiotomy reduces the incidence of supra-ventricular arrhythmias and pericardial effusion after coronary artery bypass grafting. Eur J Cardio-Thorac Surg. (2002) 22:278–81. 10.1016/S1010-7940(02)00259-2 12142199

[14] Fawzy H Elatafy E Elkassas M Elsarawy E Morsy A Fawzy A. Can posterior pericardiotomy reduce the incidence of postoperative atrial fibrillation after coronary artery bypass grafting? Interact Cardiovasc Thorac Surg. (2015) 21:488–91. 10.1093/icvts/ivv190 26188198

[15] Gaudino M Sanna T Ballman KV Robinson NB Hameed I Audisio K . Posterior left pericardiotomy for the prevention of atrial fibrillation after cardiac surgery: an adaptive, single-centre, single-blind, randomised, controlled trial. Lancet Lond Engl. (2021) 398:2075–83. 10.1016/S0140-6736(21)02490-9 34788640

[16] Haddadzadeh M Motavaselian M Rahimianfar AA Forouzannia SK Emami M Barzegar K. The effect of posterior pericardiotomy on pericardial effusion and atrial fibrillation after off-pump coronary artery bypass graft. Acta Med Iran. (2015) 53:57–61. 25597607

[17] Kaya M Iyigün T Yazici P Melek Y Göde S Güler S . The effects of posterior pericardiotomy on pericardial effusion, tamponade, and atrial fibrillation after coronary artery surgery. Kardiochirurgia Torakochirurgia Pol Pol J Cardio-Thorac Surg. (2014) 11:113–8. 10.5114/kitp.2014.43835 26336406 PMC4283862

[18] Kaya M Satilmişoglu MH Bugra AK Kyaruzi M Kafa Ü Utkusavaş A . Impact of the total pericardial closure using bilateral trap door incision and pericardial cavity intervention on outcomes following coronary artery bypass grafting: a randomized, controlled, parallel-group prospective study. Interact Cardiovasc Thorac Surg. (2015) 21:727–33. 10.1093/icvts/ivv259 26362623

[19] Kaya M Utkusavaş A Erkanli K Güler S Kyaruzi M Birant A . The preventive effects of posterior pericardiotomy with intrapericardial tube on the development of pericardial effusion, atrial fibrillation, and acute kidney injury after coronary artery surgery: A prospective, randomized, controlled trial. Thorac Cardiovasc Surg. (2016) 64:217–24. 10.1055/s-0035-1548737 25875954

[20] Kaygin MA Dag O Güneş M Senocak M Limandal HK Aslan U . Posterior pericardiotomy reduces the incidence of atrial fibrillation, pericardial effusion, and length of stay in hospital after coronary artery bypasses surgery. Tohoku J Exp Med. (2011) 225:103–8. 10.1620/tjem.225.103 21908956

[21] Kongmalai P Karunasumetta C Kuptarnond C Prathanee S Taksinachanekij S Intanoo W . The posterior pericardiotomy. Does it reduce the incidence of postoperative atrial fibrillation after coronary artery bypass grafting? J Med Assoc Thail Chotmaihet Thangphaet. (2014) 97:S97–104. 25816544

[22] Kuralay E Ozal E Demirkili U Tatar H. Effect of posterior pericardiotomy on postoperative supraventricular arrhythmias and late pericardial effusion (posterior pericardiotomy). J Thorac Cardiovasc Surg. (1999) 118:492–5. 10.1016/S0022-5223(99)70187-X 10469966

[23] Sadeghpour A Baharestani B Ghotbabady Ghasemzade B Baghaei R Givhtaje N. Influences of posterior pericardiotomy in early and late postoperative effusion of pericardium. Iran J Card Surg. (2011) 3:e8736.

[24] Zhao J Cheng Z Quan X Zhao Z. Does posterior pericardial window technique prevent pericardial tamponade after cardiac surgery? J Int Med Res. (2014) 42:416–26. 10.1177/0300060513515436 24553479

[25] Page MJ McKenzie JE Bossuyt PM Boutron I Hoffmann TC Mulrow CD . The PRISMA 2020 statement: an updated guideline for reporting systematic reviews. BMJ. (2021) 10:n71. 10.1136/bmj.n71 33782057 PMC8005924

[26] Higgins JPT Altman DG Gøtzsche PC Jüni P Moher D Oxman AD . The Cochrane Collaboration's tool for assessing risk of bias in randomised trials. BMJ. (2011) 343:d5928. 10.1136/bmj.d5928 22008217 PMC3196245

[27] Lau C Soletti GJ Olaria RP Myers P Girardi LN Gaudino M. Posterior left pericardiotomy for the prevention of atrial fibrillation after cardiac surgery. Multimed Man Cardiothorac Surg. (2021). 10.1510/mmcts.2021.083 34927401

[28] Gillinov AM Bagiella E Moskowitz AJ Raiten JM Groh MA Bowdish ME . Rate Control versus Rhythm Control for Atrial Fibrillation after Cardiac Surgery. N Engl J Med. (2016) 374:1911–21. 10.1056/NEJMoa1602002 27043047 PMC4908812

[29] Eikelboom R Sanjanwala R Le ML Yamashita MH Arora RC. Postoperative Atrial Fibrillation After Cardiac Surgery: A Systematic Review and Meta-Analysis. Ann Thorac Surg. (2021) 111:544–54. 10.1016/j.athoracsur.2020.05.104 32687821

[30] Dobrev D Aguilar M Heijman J Guichard JB Nattel S. Postoperative atrial fibrillation: mechanisms, manifestations and management. Nat Rev Cardiol. (2019) 16:417–36. 10.1038/s41569-019-0166-5 30792496

[31] Yorgancioglu C. An unusual experience with posterior pericardiotomy. Eur J Cardiothorac Surg. (2000) 18:727. 10.1016/S1010-7940(00)00586-8 11221729

[32] Bolourian AA Monfared MB Gachkar L Ghomeisi M Shahzamani M Foroughi M . The preventive effects of posterior pericardiotomy on atrial fibrillation after elective coronary artery bypass grafting. Tehran Univ Med J. (2011) 69:29–35.

