# Supplementary material for: Effect of posterior pericardiotomy in cardiac surgery: A systematic review and meta-analysis of randomized controlled trials
Source: Front Cardiovasc Med. 2022 Dec 23;9:1090102. doi: 10.3389/fcvm.2022.1090102 (PMC9816139; doi:10.3389/fcvm.2022.1090102)
Supplement: Supplementary file 1 [file Data_Sheet_1.docx]

Supplementary Material

| **Content** | **Page** |
| --- | --- |
| **Supplementary Table 1.** Search strategy applied to the Ovid MEDLINE database | 2 |
| **Supplementary Table 2.** Quality assessment of the included RCTs according to the Cochrane Collaboration’s Risk of Bias 2 (RoB 2) tool | 3 |
| **Supplementary Table 3.** Outcomes reported by each included RCT | 4 |
| **Supplementary Figure 1.** Preferred Reporting Items for Systematic Reviews and Meta-Analyses (PRISMA) flow diagram | 5 |
| **Supplementary Figure 2.** Forest plot for cardiac tamponade | 6 |
| **Supplementary Figure 3.** Forest plot for pleural effusion | 7 |
| **Supplementary Figure 4.** Leave-one-out analysis for postoperative atrial fibrillation | 8 |
| **Supplementary Figure 5.** Forest plot for operative mortality | 9 |
| **Supplementary Figure 6.** Forest plot for pulmonary complications | 10 |
| **Supplementary Figure 7.** Forest plot for need for intra-aortic balloon pump | 11 |
| **Supplementary Figure 8.** Forest plot for re-exploration for bleeding | 12 |
| **Supplementary Figure 9.** Forest plot for intensive care unit length of stay | 13 |
| **Supplementary Figure 10.** Forest plot for hospital length of stay | 14 |
| **Supplementary Figure 11.** Forest plot for chest drainage | 15 |
| **Supplementary Figure 12.** Funnel plot for the included studies | 16 |

**Supplementary Table 1.** Search strategy applied to the Ovid MEDLINE database.

**Ovid MEDLINE** (ALL – 1946 to present)

Searched on December 28, 2021

No language or article type restrictions.

| **Line #** | **Search** | **# of results** |
| --- | --- | --- |
| **1** | Pericardiectomy/ and posterior.tw | 45 |
| **2** | ((pericardiectom* or pericardiotom* or pericardotom*or pericardectom* or pericardiostom*) and (posterior)).tw | 60 |
| **3** | Pericardial Window Techniques/ or (pericardial window or pericardial fenestration*).tw | 858 |
| **4** | or/1-3 | 977 |

**Supplementary Table 2.** Quality assessment of the included RCTs according to the Cochrane Collaboration’s Risk of Bias 2 (RoB 2) tool.

| **Study** | **Type and risk level** | | | | | | |  |  |  |
| --- | --- | --- | --- | --- | --- | --- | --- | --- | --- | --- |
|  |  |  |  |  |  |  |  |  |  |  |
| Arbatli ^1^ | ? | ? | ? | ? | + | + | + |  |  |  |
| Asimakopoulos ^2^ | ? | ? | ? | ? | + | + | + |  |  |  |
| Bakhshandeh ^3^ | ? | ? | ? | ? | + | + | + |  |  |  |
| Cakalagaoglu ^4^ | ? | ? | ? | ? | + | + | + |  |  |  |
| Ekim ^5^ | ? | ? | ? | ? | + | + | + |  |  |  |
| Erdil ^6^ | + | ? | ? | ? | + | + | + |  |  |  |
| Farsak ^7^ | + | ? | ? | ? | + | + | + |  |  |  |
| Fawzy ^8^ | + | ? | ? | ? | + | + | + |  |  |  |
| Gaudino ^9^ | + | + | + | + | + | + | + |  |  |  |
| Haddadzadeh ^10^ | ? | ? | ? | ? | + | + | + |  |  |  |
| Kaya (2014) ^11^ | + | ? | + | + | + | + | + |  |  |  |
| Kaya (2015) ^12^ | + | + | + | ? | + | + | + |  |  |  |
| Kaya (2016) ^13^ | + | ? | ? | + | + | + | + |  |  |  |
| Kaygin ^14^ | ? | ? | ? | ? | + | + | + |  |  |  |
| Kongmalai ^15^ | + | + | + | + | + | + | + |  | **Key** |  |
| Kuralay ^16^ | + | ? | ? | ? | + | + | + |  | + | Low risk of bias |
| Sadeghpour ^17^ | ? | ? | ? | ? | + | + | + |  | ? | Unclear risk of bias |
| Zhao ^18^ | + | + | ? | ? | + | + | + |  | - | High risk of bias |
|  | Random sequence generation | Allocation concealment | Blinding of participants and personnel | Blinding of outcomes assessment | Incomplete outcome data | Selective reporting | Other bias |  |  |  |
|  |  |  |  |  |  |  |  |  |  |  |
|  |  |  |  |  |  |  |  |  |  |  |
|  |  |  |  |  |  |  |  |  |  |  |
|  |  |  |  |  |  |  |  |  |  |  |
|  |  |  |  |  |  |  |  |  |  |  |
|  |  |  |  |  |  |  |  |  |  |  |
|  |  |  |  |  |  |  |  |  |  |  |
|  |  |  |  |  |  |  |  |  |  |  |
|  |  |  |  |  |  |  |  |  |  |  |
|  |  |  |  |  |  |  |  |  |  |  |
|  |  |  |  |  |  |  |  |  |  |  |
|  |  |  |  |  |  |  |  |  |  |  |
|  |  |  |  |  |  |  |  |  |  |  |
|  |  |  |  |  |  |  |  |  |  |  |

**Supplementary Table 3.** Outcomes reported by each included RCT.

| Study | POAF | Early pericardial effusion | Late pericardial effusion | Operative mortality | Cardiac tamponade | Pleural effusion | Pulmonary complications | Need for IABP | Re-exploration for bleeding | ICU LOS | Hospital LOS | Chest drainage |
| --- | --- | --- | --- | --- | --- | --- | --- | --- | --- | --- | --- | --- |
| Arbatli ^1^ | ✓ | ✓ | ✓ |  | ✓ | ✓ |  | ✓ | ✓ | ✓ | ✓ |  |
| Asimakopoulos ^2^ | ✓ |  |  | ✓ |  |  |  | ✓ |  |  |  | ✓ |
| Bakhshandeh ^3^ | ✓ | ✓ | ✓ | ✓ | ✓ |  | ✓ |  | ✓ | ✓ | ✓ | ✓ |
| Cakalagaoglu ^4^ | ✓ | ✓ | ✓ | ✓ | ✓ |  | ✓ |  | ✓ | ✓ | ✓ | ✓ |
| Ekim ^5^ | ✓ | ✓ | ✓ | ✓ | ✓ | ✓ | ✓ |  | ✓ |  |  | ✓ |
| Erdil ^6^ |  | ✓ | ✓ | ✓ | ✓ | ✓ | ✓ |  | ✓ |  | ✓ | ✓ |
| Farsak ^7^ | ✓ | ✓ | ✓ |  | ✓ | ✓ | ✓ | ✓ | ✓ |  |  |  |
| Fawzy ^8^ | ✓ | ✓ |  |  | ✓ |  |  | ✓ | ✓ |  |  |  |
| Gaudino ^9^ | ✓ | ✓ | ✓ | ✓ | ✓ | ✓ | ✓ | ✓ | ✓ |  | ✓ | ✓ |
| Haddadzadeh ^10^ | ✓ | ✓ |  |  |  |  |  |  |  |  |  |  |
| Kaya (2014) ^11^ | ✓ | ✓ | ✓ | ✓ | ✓ |  | ✓ |  | ✓ |  | ✓ | ✓ |
| Kaya (2015) ^12^ | ✓ | ✓ | ✓ | ✓ | ✓ | ✓ | ✓ |  | ✓ | ✓ | ✓ | ✓ |
| Kaya (2016) ^13^ | ✓ | ✓ | ✓ | ✓ | ✓ | ✓ | ✓ | ✓ | ✓ |  | ✓ | ✓ |
| Kaygin ^14^ | ✓ | ✓ | ✓ | ✓ | ✓ | ✓ | ✓ | ✓ | ✓ |  |  | ✓ |
| Kongmalai ^15^ | ✓ | ✓ | ✓ | ✓ | ✓ | ✓ | ✓ | ✓ | ✓ | ✓ | ✓ | ✓ |
| Kuralay ^16^ | ✓ | ✓ | ✓ |  | ✓ | ✓ | ✓ |  |  |  |  |  |
| Sadeghpour ^17^ |  | ✓ | ✓ |  |  |  |  |  |  |  |  |  |
| Zhao ^18^ | ✓ | ✓ |  |  | ✓ | ✓ |  | ✓ | ✓ | ✓ |  |  |

*IABP: intra-aortic balloon pump; ICU: intensive care unit; LOS: length of stay; POAF: postoperative atrial fibrillation, RCT: randomized controlled trial.*

**Supplementary Figure 1.** Preferred Reporting Items for Systematic Reviews and Meta-Analyses (PRISMA) flow diagram.


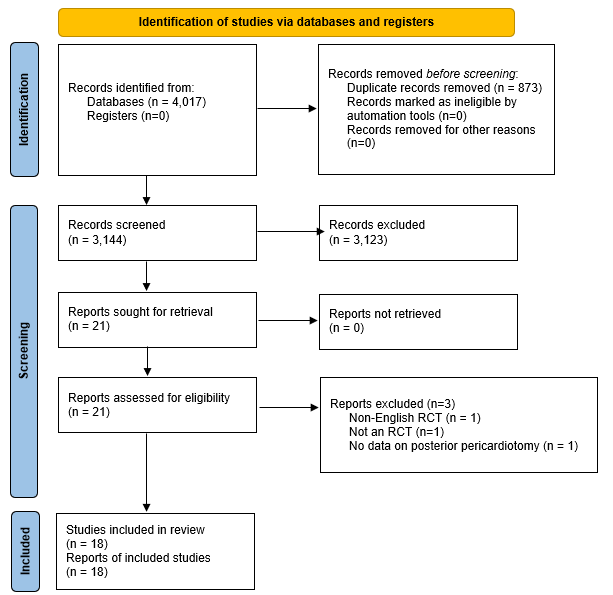


**Supplementary Figure 2.** Forest plot for cardiac tamponade. *CI: confidence interval; PP: posterior pericardiotomy; RD: risk difference.*


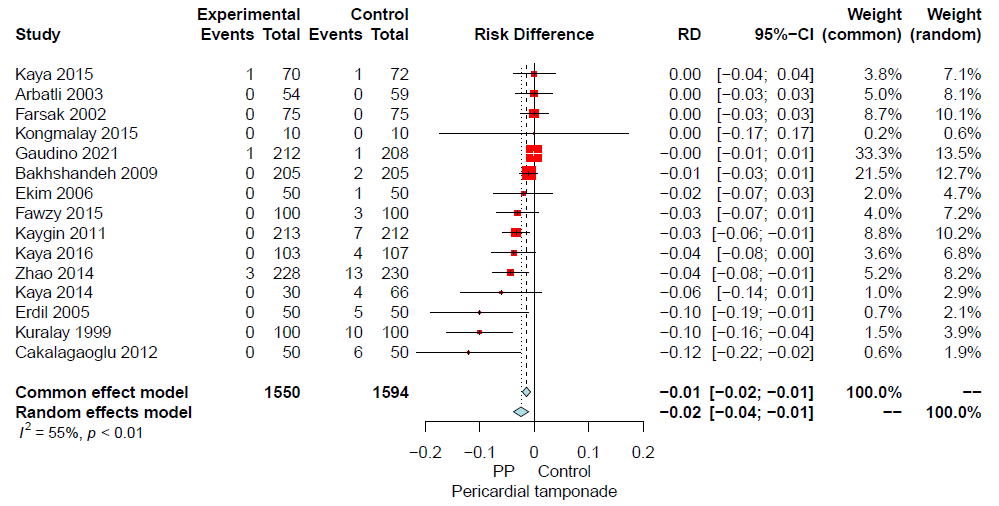


**Supplementary Figure 3.** Forest plot for pleural effusion. *CI: confidence interval; OR: odds ratio; PP: posterior pericardiotomy*.


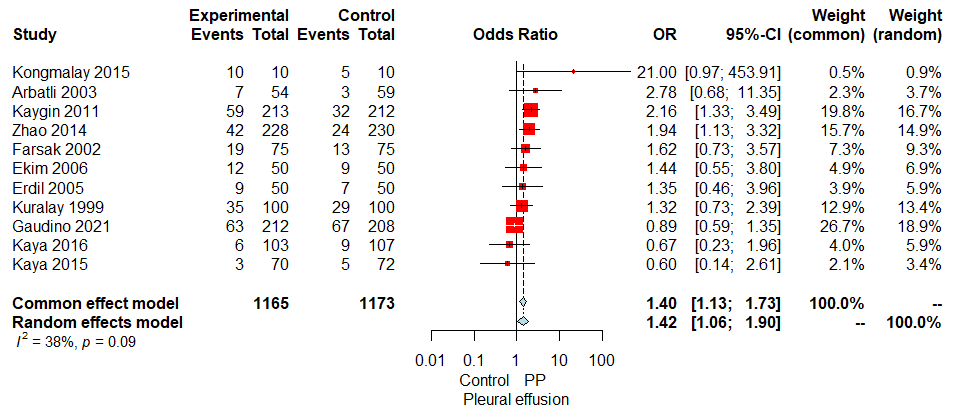


**Supplementary Figure 4.** Leave-one-out analysis for postoperative atrial fibrillation. *CI: confidence interval; OR: odds ratio; PP: posterior pericardiotomy*.


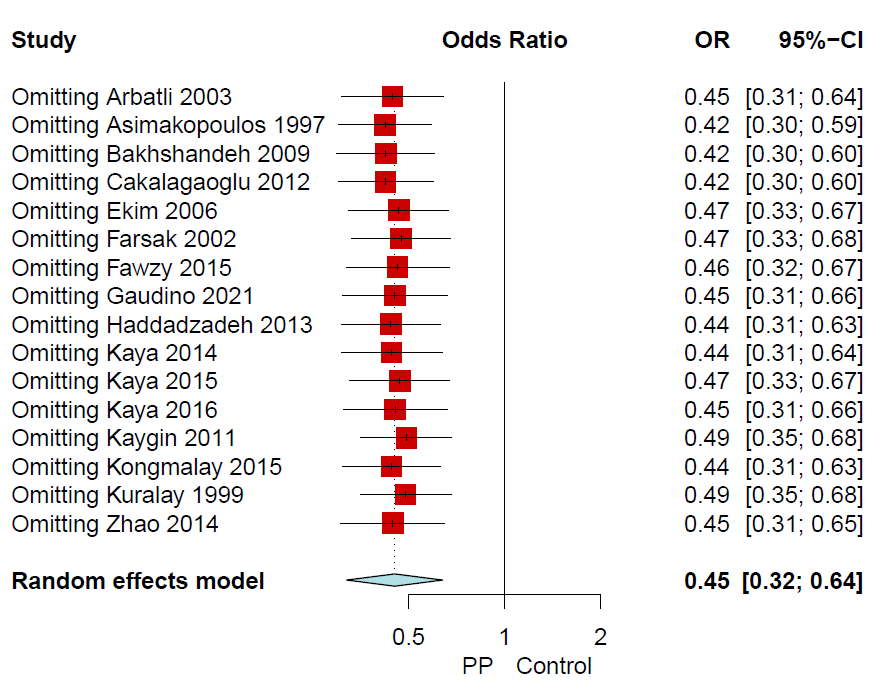


**Supplementary Figure 5.** Forest plot for operative mortality. *CI: confidence interval; PP: posterior pericardiotomy; RD: risk difference.*


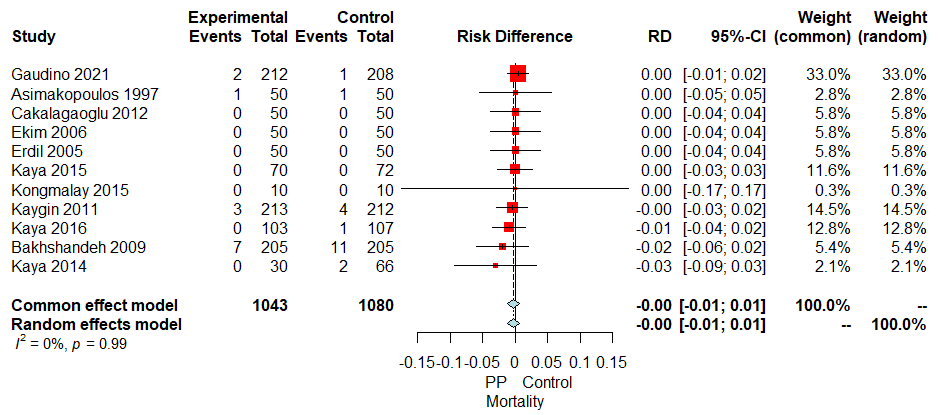


**Supplementary Figure 6.** Forest plot for pulmonary complications. *CI: confidence interval; OR: odds ratio; PP: posterior pericardiotomy*.


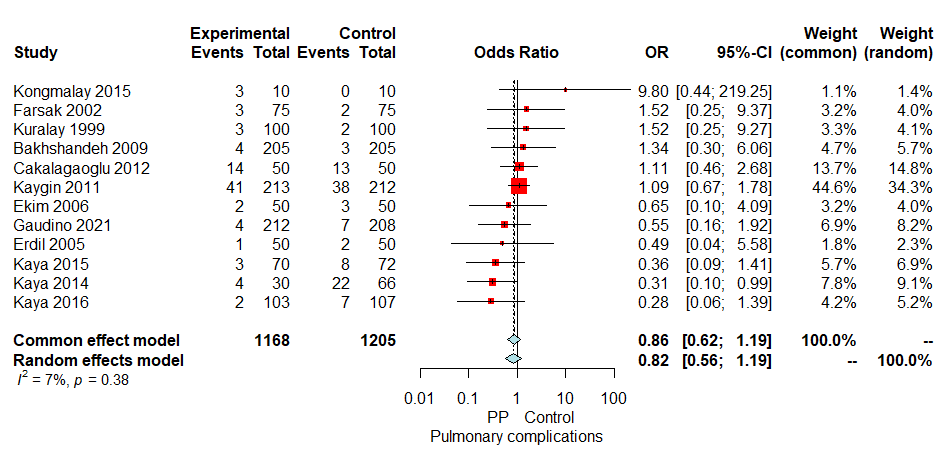


**Supplementary Figure 7.** Forest plot for need for intra-aortic balloon pump. *CI: confidence interval; IABP: intra-aortic balloon pump; PP: posterior pericardiotomy; RD: risk difference.*


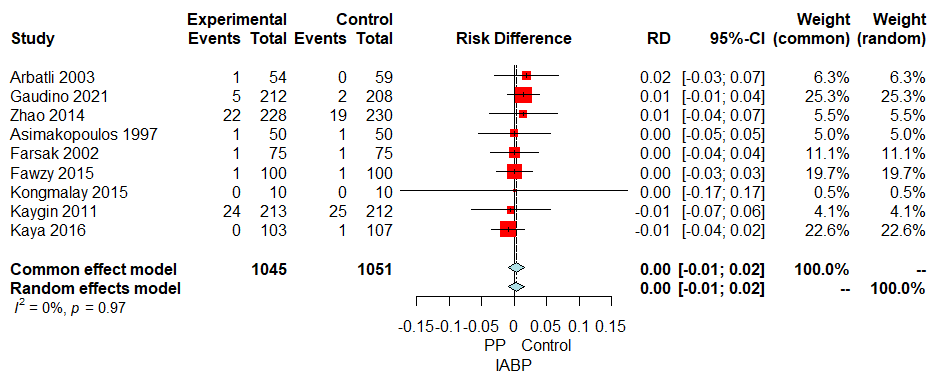


**Supplementary Figure 8.** Forest plot for re-exploration for bleeding. *CI: confidence interval; OR: odds ratio; PP: posterior pericardiotomy*.


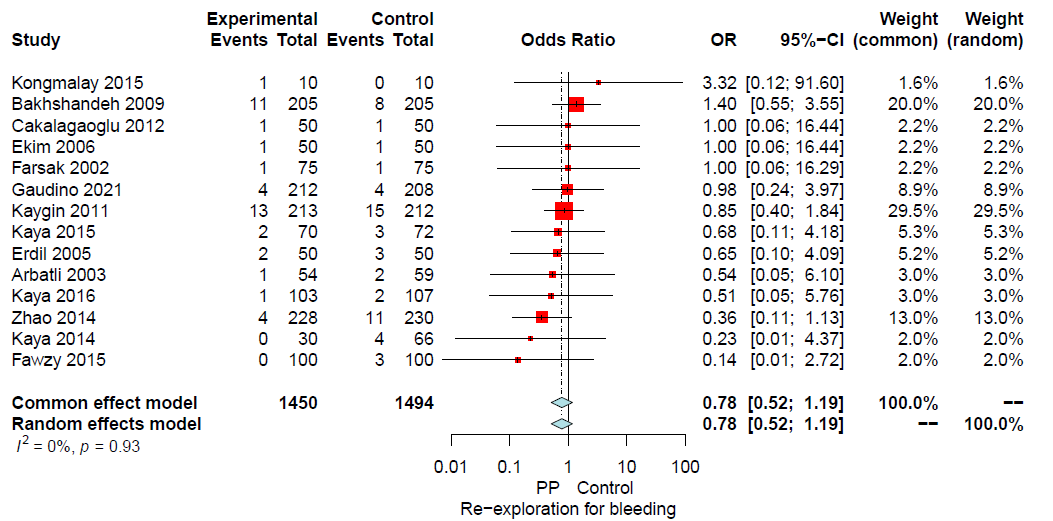


**Supplementary Figure 9.** Forest plot for intensive care unit length of stay. *CI: confidence interval; ICU: intensive care unit; LOS: length of stay; PP: posterior pericardiotomy; SMD: standardized mean difference.*


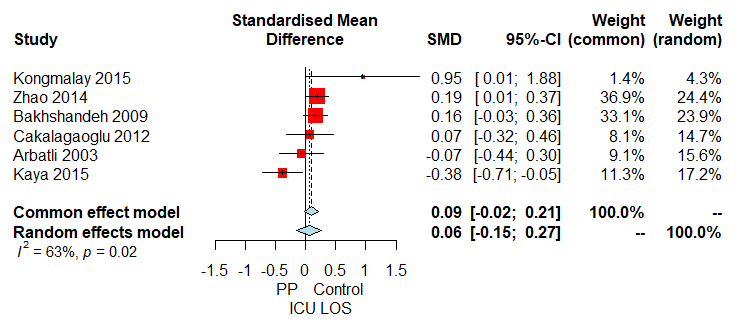


**Supplementary Figure 10.** Forest plot for hospital length of stay. *CI: confidence interval; LOS: length of stay; PP: posterior pericardiotomy; SMD: standardized mean difference.*


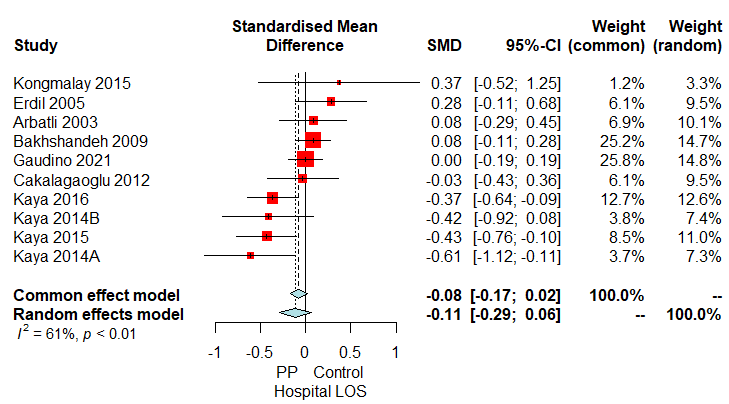


**Supplementary Figure 11.** Forest plot for chest drainage. *CI: confidence interval; PP: posterior pericardiotomy; SMD: standardized mean difference.*


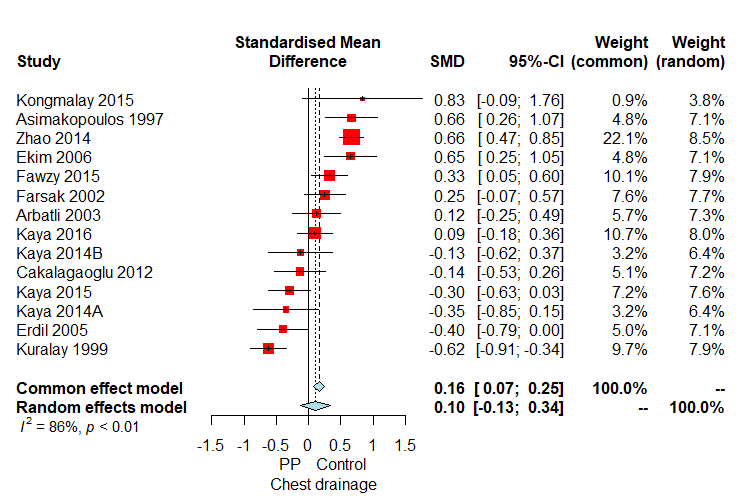


**Supplementary Figure 12.** Funnel plot for studies (n=16) reporting postoperative atrial fibrillation. Egger’s intercept test (-0.42 ± 1.27, P=0.75).


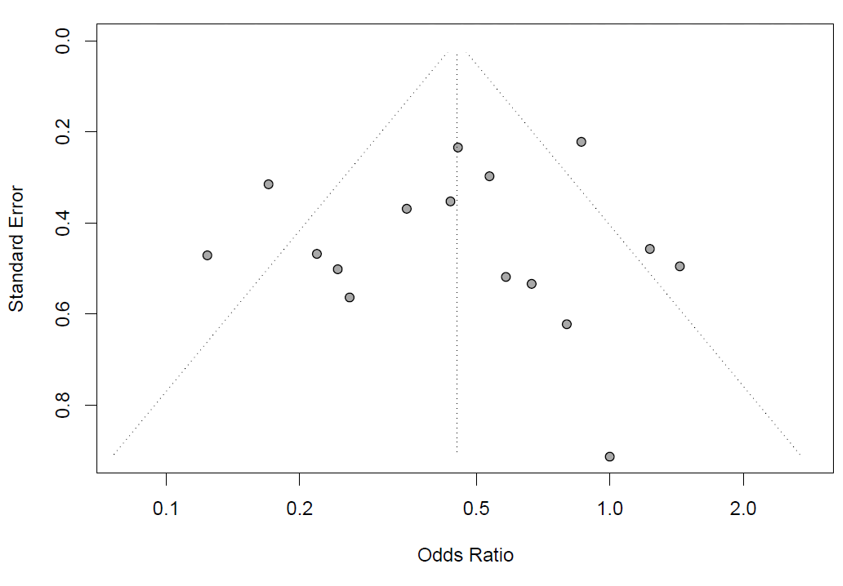


**References**

1. Arbatli H, Demirsoy E, Aytekin S, Rizaoglu E, Unal M, Yagan N, et al. The role of posterior pericardiotomy on the incidence of atrial fibrillation after coronary revascularization. J Cardiovasc Surg (Torino). 2003 Dec;44(6):713–7.

2. Asimakopoulos G, Della Santa R, Taggart DP. Effects of posterior pericardiotomy on the incidence of atrial fibrillation and chest drainage after coronary revascularization: a prospective randomized trial. J Thorac Cardiovasc Surg. 1997 Apr;113(4):797–9.

3. Bakhshandeh AR, Salehi M, Radmehr H, Sattarzadeh R, Nasr AR, Sadeghpour AH. Postoperative pericardial effusion and posterior pericardiotomy: related? Asian Cardiovasc Thorac Ann. 2009 Oct;17(5):477–9.

4. Cakalagaoglu C, Koksal C, Baysal A, Alıcı G, Ozkan B, Boyacioglu K, et al. The use of posterior pericardiotomy technique to prevent postoperative pericardial effusion in cardiac surgery. Heart Surg Forum. 2012 Apr;15(2):E84-89.

5. Ekim H, Kutay V, Hazar A, Akbayrak H, Başel H, Tuncer M. Effects of posterior pericardiotomy on the incidence of pericardial effusion and atrial fibrillation after coronary revascularization. Med Sci Monit Int Med J Exp Clin Res. 2006 Oct;12(10):CR431-434.

6. Erdil N, Nisanoglu V, Kosar F, Erdil FA, Cihan HB, Battaloglu B. Effect of posterior pericardiotomy on early and late pericardial effusion after valve replacement. J Card Surg. 2005 Jun;20(3):257–60.

7. Farsak B, Günaydin S, Tokmakoğlu H, Kandemir O, Yorgancioğlu C, Zorlutuna Y. Posterior pericardiotomy reduces the incidence of supra-ventricular arrhythmias and pericardial effusion after coronary artery bypass grafting. Eur J Cardio-Thorac Surg Off J Eur Assoc Cardio-Thorac Surg. 2002 Aug;22(2):278–81.

8. Fawzy H, Elatafy E, Elkassas M, Elsarawy E, Morsy A, Fawzy A. Can posterior pericardiotomy reduce the incidence of postoperative atrial fibrillation after coronary artery bypass grafting?†. Interact Cardiovasc Thorac Surg. 2015 Oct;21(4):488–91.

9. Gaudino M, Sanna T, Ballman KV, Robinson NB, Hameed I, Audisio K, et al. Posterior left pericardiotomy for the prevention of atrial fibrillation after cardiac surgery: an adaptive, single-centre, single-blind, randomised, controlled trial. Lancet Lond Engl. 2021 Dec 4;398(10316):2075–83.

10. Haddadzadeh M, Motavaselian M, Rahimianfar AA, Forouzannia SK, Emami M, Barzegar K. The effect of posterior pericardiotomy on pericardial effusion and atrial fibrillation after off-pump coronary artery bypass graft. Acta Med Iran. 2015;53(1):57–61.

11. Kaya M, İyigün T, Yazıcı P, Melek Y, Göde S, Güler S, et al. The effects of posterior pericardiotomy on pericardial effusion, tamponade, and atrial fibrillation after coronary artery surgery. Kardiochirurgia Torakochirurgia Pol Pol J Cardio-Thorac Surg. 2014 Jun;11(2):113–8.

12. Kaya M, Satılmışoğlu MH, Buğra AK, Kyaruzi M, Kafa Ü, Utkusavaş A, et al. Impact of the total pericardial closure using bilateral trap door incision and pericardial cavity intervention on outcomes following coronary artery bypass grafting: a randomized, controlled, parallel-group prospective study. Interact Cardiovasc Thorac Surg. 2015 Dec;21(6):727–33.

13. Kaya M, Utkusavaş A, Erkanlı K, Güler S, Kyaruzi M, Birant A, et al. The Preventive Effects of Posterior Pericardiotomy with Intrapericardial Tube on the Development of Pericardial Effusion, Atrial Fibrillation, and Acute Kidney Injury after Coronary Artery Surgery: A Prospective, Randomized, Controlled Trial. Thorac Cardiovasc Surg. 2016 Apr;64(3):217–24.

14. Kaygin MA, Dag O, Güneş M, Senocak M, Limandal HK, Aslan U, et al. Posterior pericardiotomy reduces the incidence of atrial fibrillation, pericardial effusion, and length of stay in hospital after coronary artery bypasses surgery. Tohoku J Exp Med. 2011 Oct;225(2):103–8.

15. Kongmalai P, Karunasumetta C, Kuptarnond C, Prathanee S, Taksinachanekij S, Intanoo W, et al. The posterior pericardiotomy. Does it reduce the incidence of postoperative atrial fibrillation after coronary artery bypass grafting? J Med Assoc Thail Chotmaihet Thangphaet. 2014 Oct;97 Suppl 10:S97-104.

16. Kuralay E, Ozal E, Demirkili U, Tatar H. Effect of posterior pericardiotomy on postoperative supraventricular arrhythmias and late pericardial effusion (posterior pericardiotomy). J Thorac Cardiovasc Surg. 1999 Sep;118(3):492–5.

17. Sadeghpour A, Baharestani B, Ghotbabady Ghasemzade B, Baghaei R, Givhtaje N. Influences of Posterior Pericardiotomy in Early and Late Postoperative Effusion of Pericardium. Iran J Card Surg. 2011 Jan;3(1):e8736.

18. Zhao J, Cheng Z, Quan X, Zhao Z. Does posterior pericardial window technique prevent pericardial tamponade after cardiac surgery? J Int Med Res. 2014 Apr;42(2):416–26.
